# Evaluation Effect of Shiatsu Technique on Labor Induction in Post-Term Pregnancy

**DOI:** 10.5539/gjhs.v7n3p177

**Published:** 2014-11-30

**Authors:** Teimoori Batool, Navvabi-Rigi Shahin-Dokht, Rajabi Shahnaz, Arbabisarjou Azizollah

**Affiliations:** 1 Obstetrician & Gynecologist, Promotion Health Research Center, Zahedan University of Medical Sciences, Zahedan, Iran; 2 Pregnancy Health Research Center, Zahedan University of Medical Sciences, Zahedan, Iran; 3 Obstetrician & Gynecologist, Zahedan University of Medical Sciences, Zahedan, Iran

**Keywords:** shiatsu technique, labor induction, post-term pregnancy

## Abstract

**Objective:**

Labor induction in post-term pregnancy is considered as a conventional process of mothers’ care. Shiatsu technique is one of the complementary methods which use for post-term pregnancy induction. Therefore, the researchers decided to examine the effect of Shiatsu technique on the induction of post-term pregnancy.

**Materials and Methods:**

This is a randomized control trials which conducted on 288 women with post-term pregnancy who referred to consulting clinic at Ali- Ibn- Abi -Talib Hospital, in Zahedan-Iran in 2010-2011. Participants were selected based on random table. The participants divided into two groups: the first was shiatsu technique and the second group was control group (routine procedure). Shiatsu technique was conducted on the participants of intervention group for 30s on three points by an experienced midwife. The gathered data analyzed by SPSS version 15.00 and comparing tests were t-students tests, chi-square.

**Results:**

Mothers ages range were between 16 to 42 yr (mean 26.5-5.7) in shiatsu and 17 to 43 yr (mean 24.5-5.1) in control group. Regarding spontaneous initiation of labor, 82 women (56.9%) in Shiatsu group had spontaneous initiation of labor, whereas the number of women was only 12 (8.3%) in control group. Women who have used Shiatsu technique were significantly more likely to have spontaneous labour than those women who did not.

**Conclusions:**

Results of the study showed that shiatsu technique can be used as one of safe complementary methods for post-term pregnancy induction.

## 1. Introduction

Labor induction is considered as a conventional process of mothers’ care (Hall et al., 2011). Labor induction rate in the US is mentioned as 134 out of 1000 live births and its indications include post-term pregnancy, premature rupture of amniotic sac and medical problems of mother such as diabetes and blood pressure induced during pregnancy (Summers, 1997). Standard international definition of post-term pregnancy includes pregnancy prolongation about 42 full weeks or more from the first day after the last period. Average incidence of post-term is considered as 10%. In case of one previous post-term labor the chance will be 27% and with two previous post-term labors the chance will be 39%. The chance for post-term labor recurrence will be twice or triple in daughters with post-term mothers. Minimum prenatal mortality is in 39- to 40-week age and increases after 41-week age. Low-risk pregnancies are occurring for over 42 weeks, have a high risk for prenatal morbidity and mortality (ACOG, 2004). Post-term pregnancy brings higher risk of newborns hospitalization in NICU and an increase of infancy morbidity (Norwitz, 2007). Prolonged pregnancy leads to macrosomia and difficulty in labor, and an increase in prenatal mortality (Morgen et al., 1999).

Risk factors for post-term pregnancy include primiparity, prior post-term pregnancy, male gender of the fetus, and genetic factors. Prenatal consequences in post-term pregnancy regarding prenatal mortality in 42-week age are twice 40-week pregnancies and increase quartet in 43-week and 5 to 7 times in 44-week age (Lessen et al., 1999). Post-term pregnancy is a risk factor for encephalopathy and death in the first year of life (Tzias, 1999).

A number of other post-term pregnancy risk factors are including meconium, the increase of infancy mortality rate and sudden death syndrome of infants are more wide spread in 40 week and 41 week pregnancy comparing to 39-week pregnancy (Feldman, 1992). Post-term pregnancy has also risks for the mother such as increasing labor dystocia and increased severe prenatal injuries resulted from macrosomia. This rate will be twice number of cesarean risks. The risks and side effects are higher in post-term pregnancy such as such as endometriosis, hemorrhage, thromboembolic diseases (Rand et al., 2000). Post-term pregnancy also carries a high risk of the neonates admitting to neonatal care units and is accompanied with an increased risk of obstetric and neonatal interventions (Luckas et al., 1998).

There are many methods to end post-term pregnancy including surgical, medicinal, and complementary methods which used alone or in combination with each other. The most widely used methods are herbalism induction, homeopathy, stimulating breast nipples, and acupuncture (Hall et al., 2011). Shiatsu technique is of the complementary methods use for post-term pregnancy induction. It is one of acupressure techniques. Literature reviews indicated that the validity of acupressure efficiency supports shiatsu technique efficiency. Shiatsu has been used for many years in Eastern lands as a traditional treatment method and some people remember this as Japanese physiotherapy. But now it is one of the eight complementary methods used in many eastern countries. Indeed, shiatsu makes use of energy channel and points of acupuncture medicine. The same method used in acupuncture medicine, except that here needle is not used. Unlike other massage methods, there is no need to apply oils or take off the clothes here (Ingram et al., 2005). Shiatsu is composed of two Japanese words “shi” meaning finger and “atsu” meaning pressure. Use of pressure is the main basis of shiatsu method.

Sometimes, shiatsu is mistaken with “acupressure”, whereas it is technically different from the method. Despite this method, sometimes a wide area of body is pressured in shiatsu. However, it is sometimes possible that acupuncture medicine points are pressured. A study by (Ingram et al., 2005) has been conducted on post-term pregnancy induction by shiatsu. In the study, 66 women were received massage technique from an obstetrician (who had completely done shiatsu course). Resulting information included: labor induction, type of labor, length of labor stages, weight of infant, and use of analgesia. Shiatsu points comprised point GB21 (below the top shoulder hollow), L14 (between thumb and forefinger back of the hand) and SP6 (three fingers above exterior ankle of the foot), each of which was slowly pressured so that a feeling of relaxation was felt as the reaction. The technique took no more than 15 min each session. Results showed a meaningful difference comparing to control group in post-term women subjected to shiatsu technique. Those women with Shiatsu technique, 17% more went into spontaneous labor compared to those who were not used Shiatsu technique (Ingram et al., 2005).

Use of medicinal induction has been increased since 1980 showing a ratio of 1:5 in most countries which has had dangers for mother and child, yet some women incline to complementary methods which lead the induction of labor to normal delivery (Hall et al., 2011). We decided to examine the effect of this complementary method (Shiatsu technique) on the induction of post-term pregnancy because 1). Aggressive surgical and medicinal methods have many problems for both mother and neonate; 2). Safety of shiatsu is emphasized in a review study (Robinson et al., 2011); 3). Modern medicine emphasizes on non-aggressive methods and, 4. No studies have been carried out in Iran.

## 2. Materials and Methods

This clinical trial (based on consort diagram) was finally carried out on 288 women out of 600 women referred to admittance room of Ali- Ibn- Abi -Talib Hospital affiliated to Zahedan University of Medical Sciences, Iran in 2010-2011. Participants were selected based on random table. Inclusion criteria were including reliable EDC, post-term pregnancy, non-consequence pregnancy, presentation of cephalic. Exclusion criteria were including cervix dilatation over three centimeter, active labor, and premature rupture of membranes, previous cesarean and pathology in mother or neonate. The subjects divided into two shiatsu technique and control groups (routine procedure). Sample size was determined as 170 participants based on pilot study. Shiatsu technique was conducted on the participants of intervention group for 30s on three points GB21 (below the top shoulder hollow), L14 (between thumb and forefinger back of the hand) and SP6 (three fingers above exterior ankle of the foot) by right hand thumb and using Acu-health device by an experienced midwife who had more than five years experiences at University hospital, BS in Midwifery and trained for the course. In control group, routine actions of hospital were taken. Then, necessary instructions were delivered to both groups regarding signs and symptoms of labor initiation (rupture membrane, pain and bleeding). Participants’ phone numbers were collected so that the technique was repeated in case labor would not start after 24 h. Bishop Score, mean labor duration, labor initiation, mean labor stages length, use of inductive medicines, analgesia, type of labor and fetal results indices were examined using t-students tests, chi-square through SPSS_15_ Software.

## 3. Ethical Consideration

The participants were completed written consent forms for participating in this research. They were assured that their collaboration and participation was voluntary and anonymous. All data handled with confidentiality. The ethical approach has been confirmed by the Ethics Committee of Zahedan University of Medical Sciences.

## 4. Results

**Figure 1:**
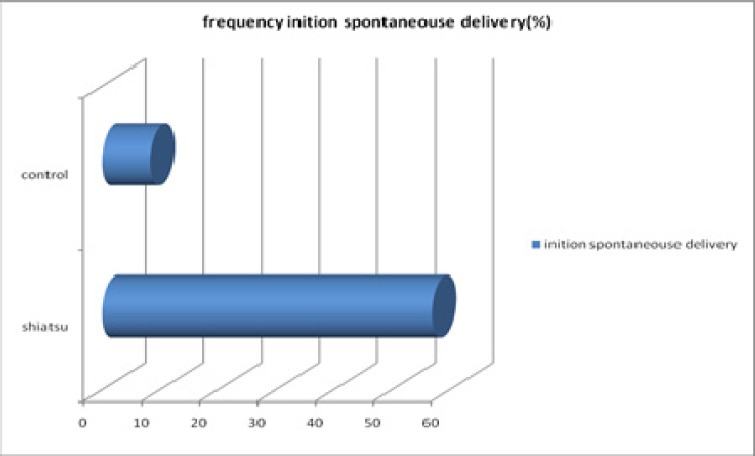
Comparison of the frequency spontaneouse delivery in two groups

**Figure 2:**
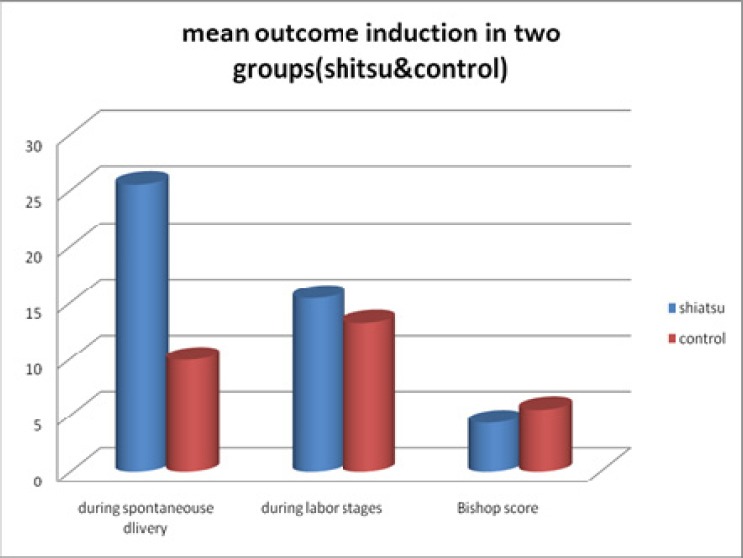
Comparison of mean outcome induction in two groups

Patients were placed in two shiatsu and control groups and age range was between 16 to 42 yr (mean 26.5-5.7) in shiatsu and 17 to 43 yr (mean 24.5-5.1) in control group. Subjects were matched regarding age. There was no meaningful differences regarding number of labors, mother age, mother job, type of delivery, and child weight. There was a meaningful difference regarding educational degree, prior post-term pregnancy, use of Oxytocin, analgesia and fetal distress. Also, statistically significant (p<0.001) difference was observed between the two groups under study (Table 1). Regarding spontaneous initiation of labor, 82 women (56.9%) in Shiatsu group had spontaneous initiation of labor, whereas the number was only 12 (8.3%) in control group (Figure 1). Regarding Bishop Score, it was 4.3 in shiatsu and 5.5 in control group both of which was above 4. Mean labor initiation duration after the first technique was 25.5 h in Shiatsu and 9.9h in control group. But mean labor stages was 15.4 h in Shiatsu and 13.2 h in control group which were not statistically significant (p>0.05) (Figure 2).

**Table 1:** Comparison variables in two shiatsu & control groups

Group characteristic	Shiatsu		control		pvalue
	
Occupation	N	(%)	N	(%)	
employed	**22 (15/3)**		**26 (18/1)**		p=0.318

House wife	**122 (84/7)**		**118 (81/9)**		

**Education level**					

Elementary/no literacy	**49 (34)**		**91 (63/2)**		p<0.001

school	**25 (17/4)**		**13 (9)**		

High school	**43 (29/9)**		**21 (14/6)**		

university	**27 (18/8)**		**19 (13/2)**		

**Previous postterm**					

Yes	**97 (67/4)**		**58 (40/3)**		p<0.001

no	**47 (32/6)**		**86 (59/7)**		

**Use oxcytocine**					

Yes	**78 (54/5)**		**134 (93/1)**		p<0.001

no	**65 (45/5)**		**10 (6/9)**		

**Use analgesia**					

Yes	**16 (38/1)**		**129 (93/5)**		p<0.001

no	**26 (61/9)**		**9 (6/5)**		

**Fetus distress**					

Yes	**5 (3/9)**		**66 (50/8)**		p<0.001

no	**124 (96/1)**		**46 (49/2)**		

**Type delivery**					

Vaginal	**119 (82/6)**		**125 (86/8)**		p<0.207

c/s	**25 (17/4)**		**19 (13/2)**		

## 5. Discussion

There are several methods for ending post-term pregnancy including surgical, medicinal and complementary methods. Shiatsu technique is one of the complementary methods used for post-term pregnancy induction. It is amongst acupressure methods. Pressure points in the technique comprise L14, SP6, and GB21.

Present study indicated the effect of this technique on spontaneous labor initiation which corresponded with (Ingram et al., 2005) study. In this research, results showed meaningful difference about post-term pregnancy and labor stages progression, spontaneous labor in comparing with control group. Participants with shiatsu technique entered into spontaneous stage 17% more than control group (Rand et al., 2000). But in present study, spontaneous labor began 48.6% more than the control group without any medicinal intervention. Perhaps, spontaneous initiation percentage is a result of integrating two methods (one is the use of hand and the other is the use of energy inducing device (acuhealth) and the time given to the samples in this study.

Here, despite other studies done in the same regard, cervix Bishop Score was higher in control group. Also mean labor initiation time and mean labor stages length in control group (routine actions) are less than shiatsu group. Only the number of spontaneous labor initiation was very higher in shiatsu group and consistent with the other studies results. Perhaps, recurrent referring and follow-up vaginal visits and striping the membranes led to the above time effect. However, not recording the frequency of the times is of research limits.

To explain the subject, we make use of other intervention variables in the study. Medicine need reduction was not examined in Ingram studies and only the type of medicine was the same. Researchers looking for non-medicinal interventions such as complementary treatments, physiotherapy, diet therapy, and consultation mostly seeking to present a design toward the stop or decrease of medicinal need, they move toward choosing benefits of treatment and reduction of medicines like painkillers. If we just measure symptoms and effects and or health status, we will remove the results or wasted them in one way or another and conclude wrongly about the efficiency of the intervention (Paterson et al., 2004).

Using cento was meaningfully higher in control group and results in sooner initiation of labor phase in this group comparing to shiatsu group. Also, use of Hyoscine, Atropine, Promethazine, and Pethidine was meaningfully higher in control group which itself was another factor for increasing Bishop Score and decreasing the labor initiation time in the group. In addition, cause of longer hospitalization of the control group patients were removing the membranes of cervix and artificial rupture which they relatively controlled by surgical operation. These were as research limitations and affected Bishop Score and labor initiation stages time. Shiatsu method has been used in Eastern countries since 5000 years ago as a treatment way. Shiatsu is composed of pressure and stretch techniques. This treatment method leads in the reduction of stress and fatigue, lymph fluid flow increase, and as a result removal of toxins from the tissues and is effective on hormonal system, immunity system and autonomic nervous system (autonomic, sympathetic, and parasympathetic). One treated by this method feel a deep relief in the end. Accordingly, analgesia reduction is explained by gate control theory in this study (Robinson et al., 2011). Methods such as pressure, acupuncture medicine, massage and shiatsu work based on gate control theory. Based on this theory, pain control hormone increases by stimulating neurons type A including two interior hard drugs, i.e., endorphin from brain and encephalin from spinal cord. When Morphine level increases, pain excitability threshold reaches a high level in the body and resulting in enhancing defense system against pain (Mccaffery & Beebe, 1994; Smeltzer SC, Bare, 2000).

Moreover, mean Apgar score was higher in shiatsu group, although, the difference was not meaningful. Also, frequency of distress was 13 times more in control group. Perhaps, it means that more interventional actions are taken in control group which must be considered. Of course, type of labor can also be among other intervention factors in studies which here there was no statistically significant changes between two groups. Researches demonstrated serious previous post-term pregnancy outcomes increase from 40 to 43 weeks in southern Iran with age (Yazdani et al., 2006).

(Lee et al., 2004) have shown in a research titled evaluation of the effect of massaging Saninjiao point on labor pain and length of labor stages that the length of labor active stage and pain severity as well as the amount of cesarean were meaningfully lower in intervention group comparing to control group. In the end, a meaningful difference was determined between labor pain and duration in both groups all the way through labor stages. The results also showed that massaging Saninjiao point is effective in reducing length of labor stages (Lee et al., 2004). However, in a study, it did not consider acupuncture as effective factor in labor induction (Harper et al., 2006).

In Lee’s study, mean Oxytocin reception was 205cc in control and 149cc in massage group and there was a meaningful difference between two groups and also in our study.78 individuals (54.5%) in shiatsu and 134 individuals (93.1%) in control group were received Oxytocin.

Ultimately the results demonstrated shiatsu technique results in increasing in the frequency of labor spontaneous initiation, decreasing in the need for Oxytocin, reduction of fetal distress and its side effects. Regarding the feasibility of its conducting, having no side effects and also being done for free, it is recommended for labor cases.

## 6. Conclusion

Shiatsu technique leads in decreasing the need for Oxytocin. Since administration of Oxytocin requires consistent monitoring of mother and fetus by experienced staff. Shiatsu technique reduces the need for Oxytocin, therefore manpower and costs are saved. Since the method does not require special time and place and is always applicable, so the research can encourage respected mangers and officials to consider the method as an efficient one and examine it in hospitals as a suitable initiative, if patients agree. Yates included that there is much potential for the integration of Shiatsu into midwifery practice (Yates, 2012). In another study, researchers found that shiatsu consultations. Participants claimed the clinic increased equality of access to complementary medicine, improved perceptions of the general practice, reduced consultation and prescription rates, enhanced GP-patient relationships and the working practices of the GPs and shiatsu practitioner. The study successfully integrated a shiatsu clinic into a general practice and offers a model for future research on complementary medicine in primary care (Zoe et al., 2012). Results of the study showed that shiatsu technique can be used as one of safe complementary methods for post-term pregnancy induction.
